# School Dropout, Absenteeism and Coverage of Sexual and Reproductive Health Services in South Africa: Are Those Most at Risk Reached?

**DOI:** 10.1007/s10461-024-04448-2

**Published:** 2024-07-19

**Authors:** Tracy McClinton Appollis, Catherine Mathews, Carl Lombard, Kim Jonas

**Affiliations:** 1 Health Systems Research Unit, South African Medical Research Council, Cape Town, South Africa; 2 Biostatistics Unit, South African Medical Research Council, Cape Town, South Africa

**Keywords:** Adolescent girls, School dropout, School absenteeism, Sexual and reproductive health risk, Combination HIV prevention, Intervention coverage

## Abstract

School attendance or completion is important for adolescents’ development. Adolescents who drop out or are regularly absent from school are at higher risk of adverse sexual and reproductive health (SRH) outcomes. However, there is little evidence evaluating SRH service coverage among adolescents in and out of school. In the context of a large-scale combination HIV and pregnancy prevention intervention funded by the Global Fund, we compared the SRH intervention coverage and SRH risks among adolescent girls who dropped out of school with those who were still in school or who had completed grade 12 in South Africa. Among those still in school, we compared the SRH intervention coverage and SRH risk profiles of those with high versus low or no absenteeism. In 2017 to 2018, we conducted a household survey of adolescent girls aged 15 to 19 years in six of the ten combination intervention districts. Of 2515 participants, 7.6% had dropped out of school. Among the 1864 participants still in school, 10.8% had high absenteeism. Ever having had sex, and condomless sex were more prevalent among dropouts compared with non-dropouts. Dropouts were more likely to access SRH services such as condoms and contraceptives, except the combination prevention intervention services which were more likely to reach those who had not dropped out and were equally likely to reach those in school with high versus low/no absenteeism. Combination SRH prevention programmes can improve the accessibility of SRH services for adolescents in school/who complete school.

## Introduction

Adolescence is a challenging time and adolescents are known to engage in risky behaviors which might lead to short-term benefits but potentially long-term harmful consequences [1]. Sexual risk behavior during adolescence is a global topic of interest and is a major public health problem [2, 3]. Behaviors such as early sexual debut, multiple sexual partners, unprotected sex, sexual activity under the influence of alcohol and drugs can lead to unintended pregnancy, STIs and HIV. Unintended pregnancies and HIV/AIDS remains a public health concern in South Africa. In South Africa, 30% of adolescent girls fall pregnant [4], with 65–71% of these being unintended [5]. Adolescent girls and young women (AGYW) aged 15–24 years account for 30% of new HIV infections in the country [6].

School truancy (staying away from school without a valid reason) during adolescence is relatively common in many parts of the world [7–10] and school dropout (leaving school before completing grade 12) is common in many low and middle-income countries [11]. Adolescents may skip classes or even end up dropping out of school for many reasons ranging from illness [7], lack of sanitary supplies during menstruation [12], mental health conditions [13], low IQ or learning difficulties [14] to bullying [13, 15], perceived lack of safety [13], exposure to violence [16], parenting problems [13], poor school climate [17], substance use [10, 18] and unreliable transportation [7].

School attendance is beneficial for a child’s development and skipping school and dropping-out has been linked to many negative consequences [19]. Evidence suggests that frequent absenteeism is detrimental and has a negative impact on children not only leading to the risk of low academic achievement [20], and eventually school dropout resulting in them struggling to find a stable job [21], leading to possible poverty, but also risky sexual and reproductive health behaviors such as early sexual debut, teenage pregnancy and HIV infection [22–24]. School dropout has also been found to be associated with higher levels of intimate partner violence (IPV) in studies conducted in India and the United States of America [25, 26]. Studies conducted in the United States of America and Taiwan found that school absenteeism, with or without permission was related to risk behaviors such as unintentional injuries and violence, substance use and risky sexual behaviors [27, 28].

Evidence suggests that attending school has positive outcomes and protects young people from adverse sexual and reproductive health (SRH) outcomes. Evidence from 9 diverse settings in sub-Saharan Africa shows that school-going adolescents between the ages of 10 and 19 years were at a lower risk for adverse health outcomes [29] compared with those out of school. In-school adolescents were less likely to report ever having had sexual intercourse (51.2%; 95% CI 45–67); more likely to have used a condom during last sex (20.5%; 95% CI 11–29); and more likely to be aware of HIV (57.6%; 95% CI 52–63) [29]. Similarly, a study conducted in rural South Africa among young women [30, 31] found that young women who stay in school and attend school regularly tend to have partners of the same age group (versus older) and fewer sexual partners than those who attend less frequently and drop out of school. Despite a lower prevalence of risk among adolescents in school, they were more likely to report an unmet need for healthcare (32.6%; 95% CI 39–91) compared with those out of school [29]. Another way in which being in school protects adolescents against negative SRH outcomes is through SRH interventions or services offered or conducted in schools. The school setting offers an appropriate environment for interventions to promote adolescent SRH, many of which have been found to be successful [32].

A review of literature has shown that dropout is more common amongst girls than boys in most countries around the world [33], this has found to be particularly true for the least wealthiest and most socially disadvantaged groups [34]. Therefore, there may be unique factors to consider which influence adolescent girls to drop out of school.

There are several studies investigating school dropout and absenteeism and their consequences and predictors, however, to our knowledge, few focus on the relationship between school dropout, absenteeism and SRH service coverage, and more specifically in South Africa. As part of an evaluation of a combination HIV and pregnancy prevention intervention, we conducted a household survey of adolescent girls aged 15 to 19 years. In these analyses, we compare the SRH risk profiles and SRH intervention coverage of those who dropped out of school before completing grade 12 with those who completed grade 12 or who were still in school. Among those still in school, we compare the SRH risk profiles and SRH intervention coverage of those with high rates of absenteeism with those with low rates or with no absenteeism. We describe and compare the following SRH risks: being HIV positive, sex without using condoms or other modern contraception, transactional sex, age-disparate sex relationships, substance use, intimate partner violence victimization and sexual violence victimization; and coverage of the following SRH interventions, services and commodities: HIV testing, access to condoms and contraceptives, emergency contraception awareness, PrEP use, online digital SRH websites and applications, economic strengthening interventions, and participation in at least one branded component of a combination HIV prevention intervention that was being implemented in the study sites.

## Methods

### Study Design and Sampling

We conducted a cross-sectional household survey as part of an evaluation of a large-scale combination HIV and pregnancy prevention intervention among adolescent girls and young women (AGYW) in ten districts within South Africa. The intervention was designed for AGYW aged 10 to 24 years and implemented from 2016 to 2019. The evaluation (named the HERStory Study) was conducted between 2017 and 2018 among 15- to 24-year-old AGYW in six of the ten districts in which the intervention was implemented. The six districts included: City of Cape Town (Western Cape), Ehlanzeni (Mpumalanga), OR Tambo (Eastern Cape), Tshwane (Gauteng), King Cetshwayo (KwaZulu-Natal), and Zululand (KwaZulu-Natal). A random sample of small area layers was selected within the geographical sub-areas in which the intervention was implemented in each district. Within each small area layer, 35% of households were randomly selected and all AGYW in the eligible age range were invited to participate. More information on the sampling can be found on [35, 36]. The analyses reported here were restricted to participants between the ages of 15 and 19 years.

### Measures

#### Socio-Demographic Indicators

##### Age was Determined by Asking Participants to Report on Both their Age and Date of Birth

*Socio-economic status (SES)* included a binary indicator which was created using a Cluster Analysis with the K-modes algorithm. Cluster Analysis is a learning technique done by a machine that allows data to be grouped based upon features they share. These features used during the creation of this indicator were: (1) AGYW was away from home for more than one month in past 12 months, (2) Has piped water in household, (3) Has flushing toilet in household, (4) Household has working electricity, (5) Household has a car, (6) Household has a computer, (7) Household has the internet, (8) Household has a refrigerator, (9) Household has a stove, (10) Went a day/night without eating in the past month, (11) AGYW has own money, (12) AGYW saves money, and (13) AGYW owes money. These variables were then grouped to indicate those with a relatively low SES and those with a high SES.

To assess whether participants *had a deceased parent* they were asked “Is your biological mother (birth mother) alive” and “Is your biological father (birth father) alive?” and given the option “yes”, “no”, and “I don’t know” to respond to.

*Length of time lived in their community* was assessed by asking “How long have you lived in this community?”, with the options “always”, “1–5 years” and “more than 5 years”.

#### In School Versus Drop Out

Participants were asked whether they were currently in school. They were also asked what their highest grade was that they had passed. When they answered that they were no longer in school and that they had not attained grade 12, they were then considered as having dropped out of school.

#### High Versus Low Absenteeism

Participants were asked whether they had been absent from school for more than one week in the past year. They were also asked how often they were absent from school in the past year with response options: “never”, “rarely (1–5 days/year)”, “occasionally (1–3 days/month)”, “regularly (1–3 days a week)” or “several weeks at a time”. They were then considered as having high absenteeism when they answered “yes” to being absent more than one week and being absent regularly or several times a week in the past year.

#### SRH Risk Indicators

*SRH Risk Indicator* questions with their response options can be found in the first column of the Tables 2 and 3. These included questions such as whether they had a boyfriend or partner in the past year, if they had ever had sex, transactional sex, dual protection, coercion, regret, partner age, and gender. Participants could answer “yes” or “no” to these questions.

The *HIV status* of AGYW were determined by laboratory results of blood samples provided by participants during the study. Two microtainers of blood were taken from each participant to use to spot DBS cards. The following tests were completed for HIV status: HIV serological testing for infection, HIV-1 ribonucleic acid (RNA) viral load measurement among those HIV serologically positive, LAg Assay to determine number and proportion of participants with recent infection. HIV seronegative was determined by screening ELISA tests and HIV seropositive on two 4th Generation ELISAs and confirmed by Western blot per HIV testing algorithm. More information on the tests performed can be found in Mathews et al. [35].

An adapted version of a questionnaire from the WHO’s multi-country study on women’s health and domestic violence against women was used for the questions on IPV [37]. Emotional, physical, and sexual violence from an intimate partner (described as “boyfriend or partner”) were measured with the use of 10-items. The questions related to *emotional IPV* asked whether their boyfriend or partner had insulted, humiliated, or threatened to hurt them. *Physical IPV* was categorized with the use of questions such as, “In the past year, has a boyfriend or partner slapped you, thrown something at you, pushed, shoved, hit, kicked, dragged, beat, choked or burned you, or used a gun or knife or nay other weapon against you?”. Questions relating to whether participants had had sex with their boyfriend or partner when they were forced, threatened or pressured were considered as *sexual IPV*. Participant’s responses were coded as “yes” that they *experienced ANY form of IPV* if they replied “yes” to emotional IPV, physical IPV or sexual IPV.

#### Substance Use

##### Alcohol use

The Alcohol Use Disorders Identification Test (AUDIT-C) is a short screening tool which contains three questions to assess problem drinking [38]. The three questions are: (1) How often do you have a drink containing alcohol? Never, monthly or less, 2–4 times a month, 2–3 times a week or > 4 times a week; (2) How many standard drinks containing alcohol do you have on a typical day when drinking? Response options range from 1 to more than 10; and (3) How often do you have 6 or more drinks on one occasion? Never, less than monthly, monthly, weekly, daily, or almost daily. A AUDIT C score of 2 or higher indicated a drinking problem. This cut-off has been validated in South Africa in another study [39].

##### Drug Use

The DUDIT was used to measure use and misuse of illegal substances, excluding alcohol and tobacco use [40]. The following questions were asked: “How often do you use drugs other than alcohol?”, “How often do you use more than one type of drug on the same occasion”, “How often are you influenced heavily by drugs”. The response options were coded with 1 = never, 2 = once a month or less often, 3 = every month, 4 = every week and 5 = daily or almost daily. If AGYW had a DUDIT score 2 or higher it is indicative of a drug use problem [41].

#### SRH Intervention Coverage

*Combination prevention Intervention Coverage* questions with their response options can be seen in the first column of the Tables 2 and 3.

*A composite measure of AGYW’s participation in intervention programmes* was created with participation measured by them having taking part in any of the following intervention programmes: Soul Buddyz Club; RISE Club; Women of Worth programme; and/or Keeping Girls in School. A description of these programmes can be found in Box 1.

*Economic strengthening support, which was provided through the combination intervention,* was measured by participants having attended a career jamboree, career day or career expo at school; received help to write a cv; or training on job interview skills from an organization in their community.

Other *SRH services* included accessing a female or male condom, accessing contraception and taking PrEP in the past year.

Box 1: Description of the combination HIV prevention for AGYW aged 10–24 years from 2016 to 2019.

**Table:** 

Name	Description
Keeping Girls in School (14–18 years)	A school-based programme which provided comprehensive SRH education, referrals to HIV testing and TB screening, linkage to care for HIV, pregnancy and other conditions, career guidance and homework support to encourage school attendance and completion of high school
Soul Buddyz Club (10–14 years)	An in-school primary school programme for children struggling academically, affected by HIV or with signs of neglect. Components included linking and referring young people to health and other services, SRH education and peer support, promoting access to grants and an environment for ongoing learning and social cohesion
RISE Clubs in- and out-of-school (15–24 years) or Women of Worth Clubs (19–24 years) (Cape Town only)	Community-based programmes that aimed to empower and build the resilience of young women and link them to biomedical services such as HIV Testing Services, prevention of mother-to-child transmission, antiretroviral therapy, modern contraceptives and other SRH services, economic strengthening and SRH education

*SBC targeted boys and girls (10–14 years-old) and therefore could not include those currently in the survey, however the young girls may have been previously exposed to the club

**Only participants in Cape Town had the opportunity to participate in the Women of Worth Programme, and this question was not asked of participants in other districts

### Study Procedures

Consent was obtained from all participants before the completion of the survey. Those under the age of 18 years were only allowed to give consent after consent from their parent/guardian/caregiver was received. The survey was conducted on an electronic device (tablet) in the participants preferred language (English, Afrikaans, isiXhosa, isiZulu, Sesotho, Sepedi, Setswana, Xitsonga). The survey was administered by a fieldworker who read the questions to the participant and entered the participants answers on the tablet. Sensitive questions regarding sexuality, SRH as well as gender-based violence and substance abuse were read by the fieldworker and the tablet was given to the participant to enter their responses privately. All surveys were conducted within a private space inside or outside the household where the participant felt comfortable.

### Statistical Analysis

We described the population by calculating frequencies (n), proportions (%) and 95% Confidence Intervals (95% CIs) for the proportions for various characteristics. We then examined bivariate relationships between variables hypothesized to influence the two different outcomes: (1) dropout status among the entire sample, and (2) high absenteeism among those who were still in school. All variables were categorical, and thus Pearson’s Chi-squared tests were used to determine if statistical differences occurred between the proportions. We also constructed logistic regression models for factors associated with the outcomes in the bivariate analyses. These models adjusted for age and relative SES in the dropout models, and only age in the high-absenteeism model because relative SES did not seem to be a confounding factor in this relationship. In these models, 15- and 16-year-olds were grouped together for the age covariate, since the bivariate analyses indicated not much difference in how these categories affected the outcomes. Adjusted odds ratios (aORs) and 95% CIs were produced for each relative measure of effect.

There was some missing data among the variables included in the analysis due to skip patterns. For example, many of the AGYW had not ever had sex, and so many questions about sexual experiences were not asked of them. Aside from this kind of missingness there were relatively few observations missing due to a preference for not responding. However, where there was missing data, we did a complete case analysis (removing missing observations). The number of missing observations is indicated in table footnotes. The analysis presented here accounts for the design of the survey by specifying the districts participants belonged to as strata, SALs as the primary sampling unit, the number of SALs in each district as the finite population correction, and sample weights.

## Results

In the HERStory dataset, there were 4399 participants. For this sub-study we excluded 1884 participants who were aged 20–24 years, resulting in a sample of 2515. Table 1 presents a brief description of the sample. There was a relatively even distribution across ages, with 18-year-olds comprising the largest fraction of the population (23.1%). Most participants belonged to the relatively low SES group (81.5%), had both parents living (56.6%), always lived in their community (71.2%), were HIV negative (93.2%) and were still in school (73.4%).

**Table 1:** Frequency distributions of participant characteristics living in South Africa (n = 2515)

	n	%	95% CI
Age			
15	508	20.0	18.8–21.2
16	469	18.3	17.1–19.5
17	492	19.6	18.4–20.8
18	581	23.1	21.7–24.6
19	465	19.1	17.8–20.3
Socio-economic status			
Relatively low socio-economic group	2100	81.5	79.9–83.1
Relatively high socio-economic group	415	18.5	16.9–20.1
Had at least one deceased parent			
No	1378	56.6	55.0–58.3
Yes	1062	40.3	38.6–41.9
Missing	75	3.1	2.6–3.7
Length of time lived in their community			
Always	1791	71.2	69.5–72.8
More than 5 years	370	15.1	13.7–16.5
1–5 years	354	13.8	12.6–15.0
HIV status^a^			
Positive	185	6.8	6.0–7.6
Negative	2329	93.2	92.4–94.0
In school and has not completed grade 12^b^			
No	647	26.6	25.2–28.1
Yes	1864	73.4	71.9–74.8

*CI* Confidence Interval

^a^One participant did not have HIV testing results available

^b^Four participants did not know the last grade they completed

There were 2323 (92.4%) participants currently in school or who had completed grade 12 and 192 who had dropped out before completing grade 12 (Table 2). Those still in school or who had graduated, compared to dropouts, tended to be younger (21.1% were 15-year-olds vs. 8.1%, p < 0.05), always lived in their community (71.6% vs. 66.9%, p < 0.05); be HIV negative (93.9% vs. 85.5%, p < 0.05); and tended not to have experienced hunger in the past month (82.5% vs. 76.7%, p < 0.05). Those who dropped out had more substance abuse, with 35.5% and 9.9% experiencing high alcohol use and high drug use respectively, compared to those who graduated or were still in school (22.7% and 5.1%, respectively, p < 0.05). Among those who dropped out 69.4% had been pregnant before, compared to only 29.4% of those still in school (p < 0.05). A relatively large fraction of the sample had experienced IPV and rape (24.0% and 6.0%, respectively), and this fraction was greater among those who dropped out (IPV: 34.3% vs. 23.1%, p < 0.05 and Rape: 14.2% vs. 5.3%, p < 0.05). In terms of sexual behavior, riskier behaviors were more common among those who had dropped out. Those who dropped out had greater prevalences of having a boyfriend in the past year (73.2%), ever having sex (77.9%), having had transactional sex (17.8%), and having a sexual partner 5 or more years older (40.4%), compared to those still in school (61.2%, 50.6%, 6.1%, and 27.4%, respectively). Those in school or who had graduated, compared to dropouts, tended to be less likely to have ever had an HIV test, less likely to have accessed male condoms or other contraception in the past year, more likely to have accessed information about sex or HIV using a phone or computer, more likely to have participated in the branded components of the combination intervention, and more likely to have received economic strengthening support (Table 2).

**Table 2:** Factors related to dropout among entire sample (n = 2515)

Variable	Overall		Dropout status						p value
			In school or attained grade 12			Dropped out			
	n	%	n	%	95% CI	n	%	95% CI	
Socio-demographic indicators									
Age									
15	508	20.0	492	21.1	19.8–22.4	16	8.1	5.1–11.2	< 0.0001
16	469	18.3	456	19.3	18.0–20.5	13	7.1	4.2–9.9	
17	492	19.6	462	19.9	18.7–21.2	30	15.2	11.1–19.3	
18	581	23.1	528	22.9	21.4–24.3	53	25.9	21.2–30.6	
19	465	19.1	385	16.9	15.7–18.0	80	43.7	38.2–49.2	
Socio-economic status									
Relatively low socio-economic group	2100	81.5	1931	81.2	79.6–82.8	169	85.4	81.3–89.4	0.0519
Relatively high socio-economic group	415	18.5	392	18.8	17.2–20.4	23	14.6	10.6–18.7	
In past month, participant or household member went a day and night without eating because of lack of food									
No	2058	82.0	1912	82.5	81.1–83.8	146	76.7	72.1–81.3	0.0196
Yes	457	18.0	411	17.5	16.2–18.9	46	23.3	18.7–27.9	
Length of time lived in their community									
Always	1791	71.2	1664	71.6	69.8–73.3	127	66.9	61.6–72.1	0.0028
More than 5 years	370	15.1	350	15.3	13.9–16.8	20	11.9	8.2–15.6	
1–5 years	354	13.8	309	13.1	11.9–14.3	45	21.2	16.7–25.8	
Had a deceased parent									
No	1378	56.6	1270	56.5	54.8–58.2	108	58.0	52.2–63.9	0.8261
Yes	1062	40.3	984	40.3	38.7–42.0	78	39.2	33.4–45.1	
Missing	75	3.1	69	3.1	2.5–3.7	6	2.7	1.1–4.3	
SRH risk indicators									
HIV status^a^									
Positive	185	6.8	154	6.1	5.3–6.8	31	14.5	10.5–18.4	< 0.0001
Negative	2329	93.2	2168	93.9	93.2–94.7	161	85.5	81.6–89.5	
Had a boyfriend or partner in the past year^a^									
No	956	37.8	905	38.8	36.9–40.7	51	26.8	21.5–32.2	< 0.0001
Yes	1524	62.2	1386	61.2	59.3–63.1	138	73.2	67.8–78.5	
Ever had sex									
No	1215	47.2	1172	49.4	47.6–51.2	43	22.1	16.8–27.4	< 0.0001
Yes	1300	52.8	1151	50.6	48.8–52.4	149	77.9	72.6–83.2	
Ever had transactional sex									
No	2336	92.9	2179	93.9	93.0–94.7	157	82.2	78.1–86.2	< 0.0001
Yes	179	7.1	144	6.1	5.3–7.0	35	17.8	13.8–21.9	
In the past year had a sexual partner 5 or more years older^a^									
No	695	71.1	631	72.6	70.3–74.9	64	59.6	53.0–66.3	< 0.0001
Yes	284	28.9	238	27.4	25.1–29.7	46	40.4	33.7–47.0	
Condom use at last sex^a^									
No	486	40.8	404	38.4	36.0–40.7	82	57.9	51.8–64.0	< 0.0001
Yes	715	59.2	656	61.6	59.3–64.0	59	42.1	36.0–48.2	
Modern contraceptive use at last sex (other than condoms)^a^									
No	837	67.3	758	68.8	66.6–71.1	79	55.9	50.2–61.6	< 0.0001
Yes	400	32.7	337	31.2	28.9–33.4	63	44.1	38.4–49.8	
Ever pregnant^a^									
No	819	65.7	778	70.6	68.6–72.5	41	30.6	25.1–36.2	< 0.0001
Yes	466	34.3	359	29.4	27.5–31.4	107	69.4	63.8–74.9	
Experienced any form of IPV									
No	1932	76.0	1805	76.9	75.3–78.5	127	65.7	60.7–70.7	< 0.0001
Yes	583	24.0	518	23.1	21.5–24.7	65	34.3	29.3–39.3	
Ever forced to have sex or raped									
No	2360	94.0	2195	94.7	94.0–95.4	165	85.8	81.5–90.1	< 0.0001
Yes	155	6.0	128	5.3	4.6–6.0	27	14.2	9.9–18.5	
Had a high alcohol use (Audit-C score 2 or higher)									
No	1963	76.3	1830	77.3	75.7–78.9	133	64.5	58.5–70.6	< 0.0001
Yes	552	23.7	493	22.7	21.1–24.3	59	35.5	29.4–41.5	
Had high drug use (Dudit score 2 or higher)									
No	2390	94.5	2213	94.9	94.1–95.7	177	90.1	86.8–93.5	0.0081
Yes	125	5.5	110	5.1	4.3–5.9	15	9.9	6.5–13.2	
SRH Intervention coverage									
Had an HIV test in the past year^a^									
No	1221	47.6	1162	48.9	47.2–50.7	59	32.7	27.5–38.0	< 0.0001
Yes	1277	52.4	1146	51.1	49.3–52.8	131	67.3	62.0–72.5	
Accessed male condoms in the past year									
No	1547	60.3	1458	61.5	59.6–63.4	89	46.4	41.4–51.5	< 0.0001
Yes	968	39.7	865	38.5	36.6–40.4	103	53.6	48.5–58.6	
Accessed female condoms in the past year									
No	2036	79.6	1884	79.8	78.3–81.3	152	77.3	72.7–82.0	0.2951
Yes	479	20.4	439	20.2	18.7–21.7	40	22.7	18.0–27.3	
Accessed contraception in the past year									
No	1819	71.6	1719	73.5	71.8–75.1	100	50.6	44.9–56.4	< 0.0001
Yes	696	28.4	604	26.5	24.9–28.2	92	49.4	43.6–55.1	
Has heard of emergency contraceptives^a^									
No	887	65.5	783	65.4	63.1–67.8	104	65.9	60.3–71.4	0.8785
Yes	452	34.5	400	34.6	32.2–36.9	52	34.1	28.6–39.7	
Has heard of PrEP^a^									
No	2281	91.4	2101	91.2	90.2–92.1	180	93.6	90.3–96.9	0.1654
Yes	209	8.6	198	8.8	7.9–9.8	11	6.4	3.1–9.7	
Has taken PrEP before^a^									
No	2457	98.4	2268	98.4	98.0–98.8	189	98.3	96.7–99.8	0.8906
Yes	40	1.6	37	1.6	1.2–2.0	3	1.7	0.2–3.3	
Has accessed information about sex or HIV using a phone or computer^a^									
No	1920	76.9	1758	76.0	74.5–77.6	162	86.3	82.5–90.0	< 0.0001
Yes	561	23.1	535	24.0	22.4–25.5	26	13.7	10.0–17.5	
Composite measure of participation Clubs, Keeping Girls at School and/or WoW interventions									
No	1151	45.6	1021	43.7	41.8–45.6	130	66.7	61.4–72.0	< 0.0001
Yes	1364	54.4	1302	56.3	54.4–58.2	62	33.3	28.0–38.6	
Economic strengthening support									
No	1621	63.7	1468	62.4	60.5–64.2	153	78.7	73.9–83.4	< 0.0001
Yes	894	36.3	855	37.6	35.8–39.5	39	21.3	16.6–26.1	

*CI* Confidence Interval

^a^Missingness: HIV status (n = 1); Boyfriend in past year (n = 35); Older sexual partner (n = 1536); Condom use at last sex (n = 1314); Contraceptive use at last sex (n = 1278); Ever pregnant (n = 1230); HIV test (n = 17); Heard of emergency contraceptives (n = 1176); Heard of PrEP (n = 25); Taken PrEP (n = 18); Accessed information about HIV or sex (n = 34)

Among the 1864 participants who were still in school who had not yet completed grade 12, there were 1663 (89.2%) participants classified as having low absenteeism and 201 with high absenteeism (Table 3). A greater fraction of those with low absenteeism were young: 26.5% were 15-years old and 23.3% were 16-years old, compared to 20.8% and 17.3% of those with high absenteeism, respectively (p < 0.05). Of those with high absenteeism, 10.2% were HIV positive versus only 5.5% of those with low absenteeism (p < 0.05). Those with low school absenteeism had lower prevalences of experiencing hunger in the past month (17.4% vs 26.2%, p < 0.05) and reporting high alcohol use (19.4% vs. 30.1%, p < 0.05) compared to those who were absent frequently. Having ever had a pregnancy was still fairly common among those in school (25.9%), however was more frequent among those who had high absenteeism (33.7% vs. 24.3%, p < 0.05). Adolescent girls who had high absenteeism also experienced more IPV (30.3% vs. 20.1%) and rape (9.6% vs. 5.0%) compared to those who had low absenteeism (p < 0.05, respectively). Smaller proportions of those with low absenteeism had a boyfriend or partner in the past year (57.4% vs. 71.9%), ever had sex (43.7% vs. 66.4%), ever had transactional sex (5.8% vs. 9.4%) and had an older partner (22.9% vs. 32.1%) compared to AGYW with high absenteeism. With regards to intervention coverage, compared with participants with low or no absenteeism, those with high absenteeism were more likely to have had an HIV test (63.6%), accessed a male condom (46.5%) and female condom (23.8%), heard of emergency contraceptives (36.6%) in the past year, accessed contraception in the past year (33.2%), and accessed information about sex or HIV using a phone or computer (25.6%) compared to those with low absenteeism (45.8%, 33.0%, 17.4%, 27.4%, 23.1%, and 20.45 respectively). We also found that more AGYW with high absenteeism accessed information about sex or HIV (25.6%) than those with low absenteeism (20.4%). There were no significant associations between accessing a component of the combination intervention or the economic strengthening support and absenteeism.

**Table 3:** Factors related to absenteeism among those who were still in school (n = 1864)

Variable	Overall		Absenteeism status^a^						p value
			Low absenteeism			High absenteeism			
	n	%	n	%	95% CI	n	%	95% CI	
Socio-demographic indicators									
Age									
15	480	25.8	439	26.5	24.7–28.2	41	20.8	16.2–25.3	< 0.0001
16	431	22.6	395	23.3	21.7–25.0	36	17.3	13.4–21.2	
17	402	21.6	363	21.9	20.3–23.4	39	19.6	15.4–23.9	
18	367	20.2	315	19.2	17.6–20.7	52	27.5	22.5–32.6	
19	184	9.8	151	9.1	8.1–10.2	33	14.8	11.2–18.3	
Socio-economic status									
Relatively low socio-economic group	1560	82.3	1387	82.2	80.6–83.9	173	82.5	77.4–87.5	0.9327
Relatively high socio-economic group	304	17.7	276	17.8	16.1–19.4	28	17.5	12.5–22.6	
In past month, participant or household member went a day and night without eating because of lack of food									
No	1518	81.6	1369	82.6	81.1–84.1	149	73.8	68.9–78.6	< 0.0001
Yes	346	18.4	294	17.4	15.9–18.9	52	26.2	21.4–31.1	
Length of time lived in their community									
Always	1326	71.0	1176	70.5	68.5–72.5	150	74.5	69.9–79.2	0.2271
More than 5 years	285	15.6	261	16.0	14.3–17.6	24	13.0	9.5–16.5	
1–5 years	253	13.4	226	13.5	12.2–14.9	27	12.5	8.8–16.1	
Had a deceased parent									
No	1012	56.1	895	55.5	53.6–57.4	117	60.6	55.3–65.9	0.1858
Yes	793	40.7	715	41.3	39.4–43.1	78	36.1	30.8–41.4	
Missing	59	3.2	53	3.2	2.6–3.9	6	3.3	1.3–5.4	
SRH risk indicators									
HIV status^b^									
Positive	122	6.0	97	5.5	4.7–6.3	25	10.2	7.3–13.1	0.0024
Negative	1741	94.0	1565	94.5	93.7–95.3	176	89.8	86.9–92.7	
Had a boyfriend or partner in the past year^b^									
No	771	40.9	712	42.6	40.5–44.6	59	28.1	23.3–33.0	< 0.0001
Yes	1066	59.1	929	57.4	55.4–59.5	137	71.9	67.0–76.7	
Prefer Not to Say	0	0.0	0	0.0	0.0–0.0	0	0.0	0.0–0.0	
Ever had sex									
No	1023	53.7	954	56.3	54.2–58.3	69	33.6	28.4–38.8	< 0.0001
Yes	841	46.3	709	43.7	41.7–45.8	132	66.4	61.2–71.6	
Ever had transactional sex									
No	1748	93.8	1568	94.2	93.3–95.2	180	90.6	87.3–93.9	0.0367
Yes	116	6.2	95	5.8	4.8–6.7	21	9.4	6.1–12.7	
In the past year had a sexual partner 5 or more years older^b^									
No	473	75.6	408	77.1	74.1–80.1	65	67.9	59.0–76.9	0.0756
Yes	146	24.4	121	22.9	19.9–25.9	25	32.1	23.1–41.0	
Condom use at last sex^b^									
No	300	38.8	250	38.0	35.0–41.0	50	43.0	35.4–50.5	0.2296
Yes	479	61.2	411	62.0	59.0–65.0	68	57.0	49.5–64.6	
Prefer not to say	0	0.0	0	0.0	0.0–0.0	0	0.0	0.0–0.0	
Missing	0	0.0	0	0.0	0.0–0.0	0	0.0	0.0–0.0	
Modern contraceptive use at last sex (other than condoms)^b^									
No	547	68.4	472	69.9	67.1–72.6	75	61.1	54.3–67.9	0.0179
Yes	251	31.6	201	30.1	27.4–32.9	50	38.9	32.1–45.7	
Missing	0	0.0	0	0.0	0.0–0.0	0	0.0	0.0–0.0	
Ever pregnant^b^									
No	596	74.1	517	75.7	73.4–77.9	79	66.3	59.9–72.7	0.0063
Yes	235	25.9	183	24.3	22.1–26.6	52	33.7	27.3–40.1	
Prefer Not to Say	0	0.0	0	0.0	0.0–0.0	0	0.0	0.0–0.0	
Experienced any form of IPV									
No	1483	78.7	1341	79.9	78.2–81.6	142	69.7	64.9–74.6	< 0.0001
Yes	381	21.3	322	20.1	18.4–21.8	59	30.3	25.4–35.1	
Ever forced to have sex or raped									
No	1755	94.4	1574	95.0	94.2–95.8	181	90.4	87.5–93.2	0.0042
Yes	109	5.6	89	5.0	4.2–5.8	20	9.6	6.8–12.5	
Had an high alcohol use (Audit-C score 2 or higher)									
No	1506	79.4	1359	80.6	79.0–82.2	147	69.9	64.1–75.7	< 0.0001
Yes	358	20.6	304	19.4	17.8–21.0	54	30.1	24.3–35.9	
Had high drug use (Dudit score 2 or higher)									
No	1785	95.3	1594	95.6	94.8–96.4	191	93.4	90.4–96.4	0.1697
Yes	79	4.7	69	4.4	3.6–5.2	10	6.6	3.6–9.6	
SRH intervention coverage									
Had an HIV test in the past year^b^									
No	993	52.1	920	54.2	52.1–56.2	73	36.4	31.0–41.7	< 0.0001
Yes	860	47.9	734	45.8	43.8–47.9	126	63.6	58.3–69.0	
Prefer Not to Say	0	0.0	0	0.0	0.0–0.0	0	0.0	0.0–0.0	
Accessed male condoms in the past year									
No	1244	65.4	1132	67.0	65.0–69.0	112	53.5	47.8–59.2	< 0.0001
Yes	620	34.6	531	33.0	31.0–35.0	89	46.5	40.8–52.2	
Accessed female condoms in the past year									
No	1548	81.9	1392	82.6	81.1–84.2	156	76.2	71.3–81.2	0.0176
Yes	316	18.1	271	17.4	15.8–18.9	45	23.8	18.8–28.7	
Accessed contraception in the past year									
No	1427	75.7	1296	76.9	75.0–78.8	131	66.8	61.4–72.1	< 0.0001
Yes	437	24.3	367	23.1	21.2–25.0	70	33.2	27.9–38.6	
Has heard of emergency contraceptives^b^									
No	617	71.1	529	72.6	69.9–75.2	88	63.4	56.8–70.1	0.0148
Yes	246	28.9	201	27.4	24.8–30.1	45	36.6	29.9–43.2	
Prefer Not to Say	0	0.0	0	0.0	0.0–0.0	0	0.0	0.0–0.0	
Has heard of PrEP^b^									
No	1702	92.1	1519	92.0	90.9–93.1	183	92.8	90.1–95.6	0.5898
Yes	144	7.9	130	8.0	6.9–9.1	14	7.2	4.4–9.9	
Prefer Not to Say	0	0.0	0	0.0	0.0–0.0	0	0.0	0.0–0.0	
Has taken PrEP before^b^									
No	1822	98.7	1633	98.8	98.4–99.2	189	97.2	95.6–98.9	0.0545
Yes	25	1.3	19	1.2	0.8–1.6	6	2.8	1.1–4.4	
Prefer Not to Say	0	0.0	0	0.0	0.0–0.0	0	0.0	0.0–0.0	
Has accessed information about sex or HIV using a phone or computer^b^									
No	1459	79.0	1312	79.6	78.0–81.2	147	74.4	69.7–79.2	0.0328
Yes	382	21.0	333	20.4	18.8–22.0	49	25.6	20.8–30.3	
Prefer Not to Say	0	0.0	0	0.0	0.0–0.0	0	0.0	0.0–0.0	
Composite measure of participation Clubs, Keeping Girls at School and/or WoW interventions									
No	795	41.7	716	42.0	39.8–44.2	79	39.5	33.7–45.3	0.4059
Yes	1069	58.3	947	58.0	55.8–60.2	122	60.5	54.7–66.3	
Economic strengthening support									
No	1239	65.7	1100	65.6	63.4–67.8	139	66.0	60.5–71.5	0.8964
Yes	625	34.3	563	34.4	32.2–36.6	62	34.0	28.5–39.5	

*CI* Confidence Interval

^a^High absenteeism was defined as those who said they were absent from school, either: 1–3 times per week, several weeks at a time, or absent for more than 1 week in the school year

^b^Missingness: HIV status (n = 1); Boyfriend in past year (n = 27); Older sexual partner (n = 1245); Condom use at last sex (n = 1085); Contraceptive use at last sex (n = 1066); Ever pregnant (n = 1033); HIV test (n = 11); Heard of emergency contraceptives (n = 1001); Heard of PrEP (n = 18); Taken PrEP (n = 17); Accessed information about HIV or sex (n = 23)

**Fig. 1:**
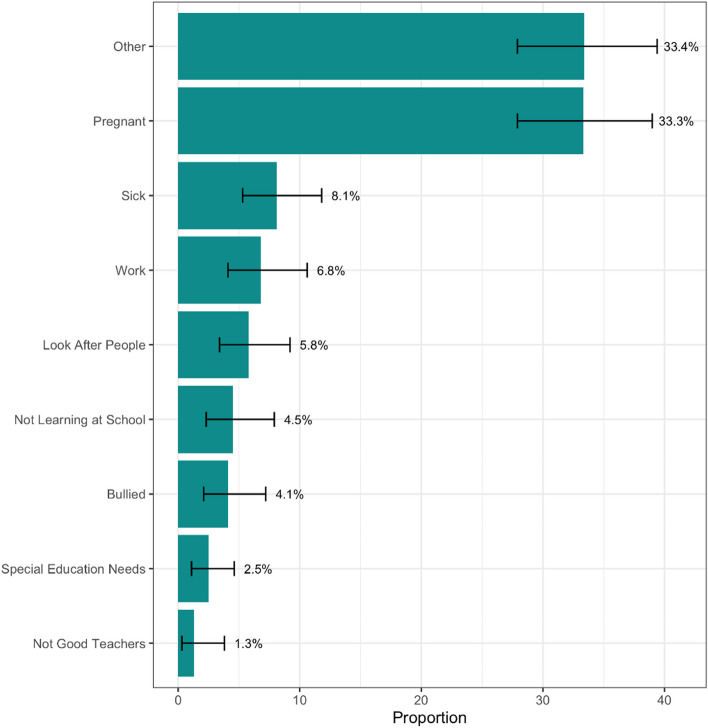
Reasons provided for leaving school among those who dropped out (n = 179). Among the original 192 who said they were not currently in school and they had not completed grade 12, 13 were also excluded from this figure for contradictory information on this question

**Fig. 2:**
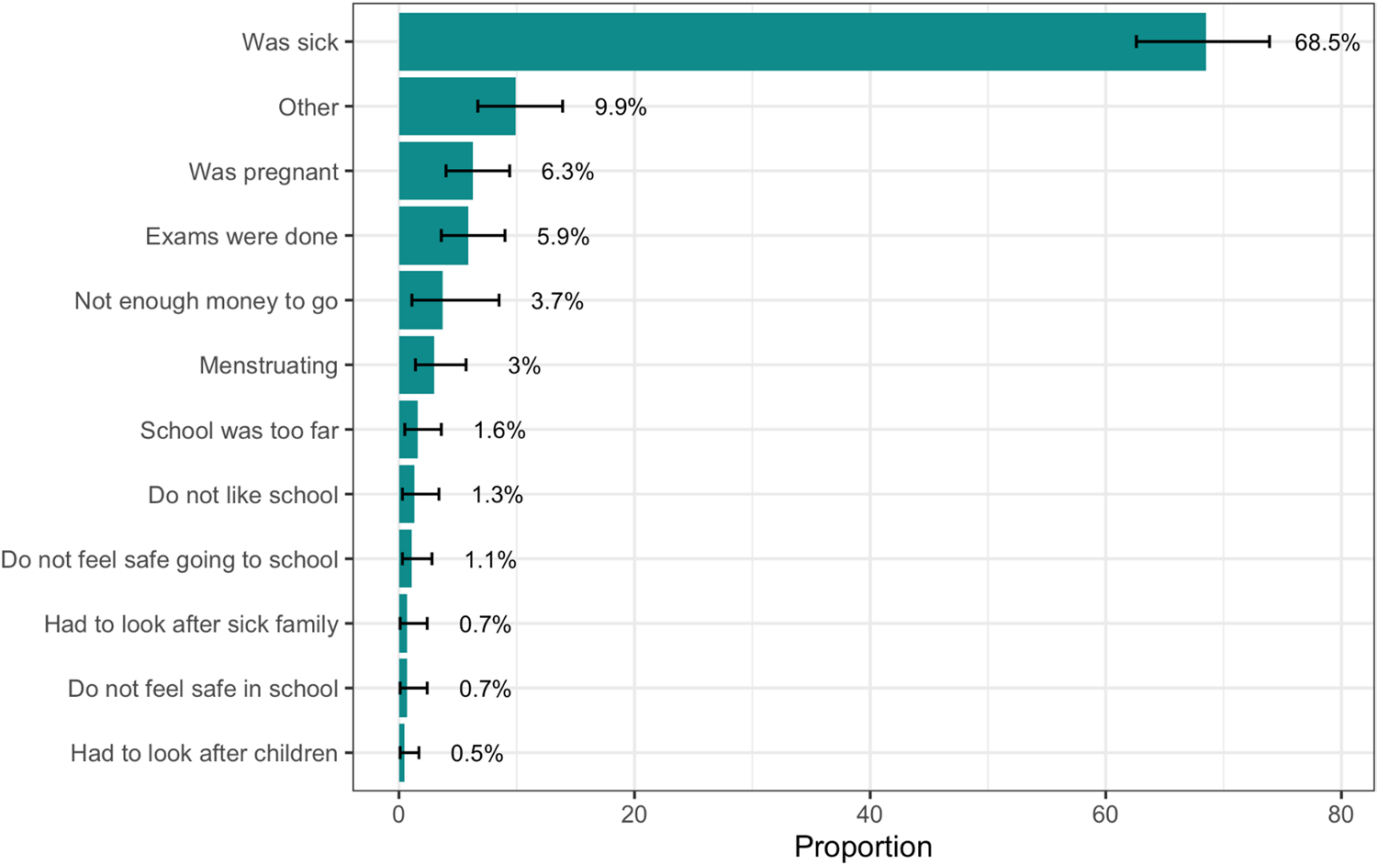
Reasons provided for being absent from school, among those who said they were they were absent from school for more than 1 week in the previous year (n = 166). 35 participants who were classified as having high absenteeism were excluded from this figure because they did not get asked this question. Participants could pick multiple reasons

Reasons AGYW dropped out of school are depicted in Fig. 1. By far the two most cited reasons for leaving school were that they had gotten pregnant (33.3%) and other reasons not listed in the questionnaire (33.4%). Leaving school because they were sick or had to work were also common (8.1% and 6.8%, respectively). AGYW who were still in school also provided typical reasons for being absent from school (Fig. 2). Among those who were absent from school for more than one week in the previous year, 68.5% said they had been sick. Factors related to school itself (e.g. not feeling safe being or going to school) were not commonly cited reasons for being absent.

### Age-Adjusted Comparisons

Table 4 shows estimates of the relationship between significant variables associated with school dropout and absenteeism. After adjusting for age and relative SES, all of the relationships remain significant in the dropout models. Adolescents who had dropped out of school had higher odds of reporting household hunger, having lived in their community for 1–5 years, having a boyfriend in the past year, having ever had sex, having transactional sex, living with HIV, having an age disparate partnership, having not used a condom at last sex, using contraceptives at last sex, having ever been pregnant, having ever been raped or experienced IPV, and having high alcohol and drug use compared to those still in school or who had completed school (Table 4). Regarding access to preventive interventions, after adjusting for age and SES, adolescent girls who had dropped out of school had higher odds of having accessed a form of contraception in the past year, having a HIV test in the past year, and having accessed male condoms in the past year compared to those who were still in school or had completed school (Table 4). However, those who had dropped out of school were less likely to have accessed a component of the combination prevention intervention, accessed information about sex or HIV from a phone or computer and participated in the economic strengthening component of the combination intervention compared with those who were still in school or had graduated, after adjusting for age and SES (Table 4).

**Table 4:** Effect estimates for relationship between SRH risk indicators, dropout and absenteeism in South Africa

Variables	Dropout	High absenteeism
	aOR (95% CI)	aOR (95% CI)
In past month, participant or household member went a day and night without eating because of lack of food		
No	Ref	Ref
Yes	1.39 (1.04–1.85)	1.68 (1.29–2.19)
Length of time lived in their community		
Always	Ref	–
More than 5 years	0.80 (0.56–1.16)	–
1–5 years	1.61 (1.18–2.19)	–
HIV status		
Positive	Ref	Ref
Negative	0.51 (0.36–0.73)	0.59 (0.40–0.85)
Had a boyfriend or partner in the past year		
No	Ref	Ref
Yes	1.42 (1.07–1. 87)	1.76 (1.38–2.24)
Ever had sex		
No	Ref	Ref
Yes	2.27 (1.58–3.24)	2.29 (1.78–2.93)
Ever had transactional sex		
No	Ref	Ref
Yes	3.22 (2.34–4.44)	1.64 (1.07–2.49)
In the past year had a sexual partner 5 or more years older		
No	Ref	–
Yes	1.84 (1.41–2.41)	–
Used a condom at last sex		
No	Ref	–
Yes	0.44 (0.34–0.58)	–
Used contraceptives at last sex		
No	Ref	Ref
Yes	1.56 (1.21–2.00)	1.40 (1.03–1.90)
Ever pregnant		
No	Ref	Ref
Yes	4.60 (3.34–6.34)	1.49 (1.08–2.06)
Experienced any form of IPV		
No	Ref	Ref
Yes	1.51 (1.18–1.92)	1.61 (1.25–2.08)
Ever forced to have sex or raped		
No	Ref	Ref
Yes	2.80 (1.91–4.10)	1.87 (1.29–2.73)
Had a high alcohol use		
No	Ref	Ref
Yes	1.57 (1.18–2.08)	1.70 (1.27–2.27)
Had high drug use		
No	Ref	–
Yes	2.30 (1.48–3.58)	–
Had an HIV test in past year		
No	Ref	Ref
Yes	1.46 (1.13–1.88)	1.89 (1.48–2.41)
Accessed male condoms in past year		
No	Ref	Ref
Yes	1.28 (1.02–1.59)	1.57 (1.24–2.00)
Accessed female condoms in past year		
No	–	Ref
Yes	–	1.38 (1.03–1.85)
Accessed a form of contraception in past year		
No	Ref	Ref
Yes	1.97 (1.52–2.55)	1.46 (1.13–1.88)
Has heard of emergency contraceptives		
No	–	Ref
Yes	–	1.52 (1.13–2.06)
Has taken PrEP		
No	–	Ref
Yes	–	2.33 (1.20–4.50)
Accessed information about sex or HIV from phone or computer		
No	Ref	Ref
Yes	0.45 (0.32–0.63)	1.31 (1.02–1.69)
Accessed a component of the intervention		
No	Ref	–
Yes	0.41 (0.32–0.52)	–
Engaged in economic strengthening support		
No	Ref	–
Yes	0.41 (0.32–0.55)	–

Each dropout model is adjusted for age and relative SES, while each absenteeism model is adjusted for only age

*aOR* Adjusted Odds Ratio, *CI* Confidence Interval

Among adolescent girls still in school, after adjusting for age, the relationship between significant variables associated with high absenteeism also remained (Table 4). Adolescents who had higher absenteeism were more likely to report household hunger, living with HIV, having a boyfriend in the previous year, having ever had sex, having ever had transactional sex, having ever been raped or experienced IPV, having high alcohol use, and having ever been pregnant compared with those who reported lower absenteeism (Table 4). Regarding coverage of interventions, those who had been absent from school often had higher odds of having had an HIV test in the past year, used contraceptives at their last sex, accessed a form of contraception in the past year, accessed a male and female condom in the past year, accessed information about sex or HIV from a phone or computer, heard about emergency contraceptives, and ever taken PrEP compared to those who were in school often, after adjusting for age (Table 4). However, there were no associations between accessing a component of the combination intervention and absenteeism.

## Discussion

The first objective of this study was to compare the SRH risk profiles and SRH intervention coverage of girls who dropped out of school with those who were still in school or completed school. Results from this study show that dropping out of school had a higher association with SRH risk factors than staying in school. While the second objective of this study was to compare the SRH risk profiles and SRH intervention coverage of those with high absenteeism to those with low absenteeism among those still in school. We similarly found that participants with high absenteeism reported riskier sexual behavior compared with those with low absenteeism.

The population of AGYW in this study lived in some of the poorest, most disadvantaged, and most HIV affected communities in South Africa, which had been purposively selected to receive the donor funded combination HIV and pregnancy prevention intervention that is the subject of this study. The overwhelming majority of the participants were in the lower SES group, highlighting the vulnerability of the study population.

In relation to the first objective, AGYW who dropped out of school had a greater prevalence of ever having had sex than those still in school. Bivariate analysis also show that they were 2 times more likely to drop out if they had ever had sex than those who never had sex. This finding is supported by a large study conducted in 9 diverse areas within sub-Saharan Africa, where adolescents still in school were 51.2% less likely (95% CI 45–67) than those out of school to report ever having had sexual intercourse [29]. Evidence suggests that school attendance may prevent SRH risks by providing times of structure and supervision in young people’s lives and therefore reducing opportunities for sexual activity [30, 31, 42].

Transactional sex was also found to have a strong relationship with participants having dropped out of school with AGYW who had transactional sex to be 3 times more likely to have dropped out of school. No other studies were found which looked at transactional sex and school dropout. This relationship between transactional sex and drop out needs further investigation.

Age-disparate relationships were found to be associated with school drop-out in this study as well as in other studies conducted in South Africa [30, 31]. Studies suggest that young women who stay in school tend to have partners closer to their age group and that this might be due to the safer sexual network structure and health literacy that school provides [30, 31, 43, 44]. Also, school provides time constraints, so the more time spent in school, the less time adolescents have for sexual activity and to spend with those outside their age group [30, 31].

One of the main reasons cited for leaving school among participants were found to be having fallen pregnant. Participants who were pregnant before were also almost 5 times more likely to drop out of school than those who had never been pregnant. This finding was corroborated in two reviews, one conducted on the factors causing dropout in girls (Shahidul 2015) and another on the prevalence and determinants of adolescent pregnancy in Africa [45], as well as studies in South Africa [46], in Nigeria [47], Kenya [48] and Malawi [49]. A study conducted in KwaZulu-Natal, South Africa, examining factors associated with school girl pregnancy found that young women will drop out of school after a pregnancy especially when she is the only the primary caregiver, than those who have help with childcare responsibilities [22]. This would most likely be the case for participants in our study as well with most of our AGYW belonging to the relatively low SES group and probably unable to afford childcare. We are however unable to confirm this as more specific questions relating to childcare were not included in our survey.

A larger percentage of participants who dropped out of school experienced IPV and rape compared to those in school, with those who had been raped being almost 3 times more likely to drop out of school than those who had not been raped. A study conducted in the Democratic Republic of Congo with school children who dropped out, found that 8% of females reported unwanted pregnancy, rape or early marriage as the reason for dropping out [50]. To our knowledge, no studies relating to IPV or rape and drop out were found within South Africa besides ours. Further research is needed around this topic to understand why this is the case. Perhaps victims do not receive counseling to cope with the rape or IPV and therefore cannot cope in school and drop out.

Participants who dropped out of school reported more substance abuse such as alcohol and drugs compared to those who graduated or completed school. Drug use particularly was found to have a strong relationship with dropout, with higher drug users being more likely to dropout than lower drug users. Fernandez-Suarez’s et al. (2016) findings support this finding by stating that substance abuse and dropout has been studied extensively, showing that substance use is associated with a higher risk of dropping out of school [51, 52]. This finding supports the perception that when students are not in school where there is structured time for most of the day, they have more time for other risky behaviors and suggests that school attendance provides a protective effect against adverse SRH outcomes, and many other studies support this finding [29, 53].

Our study found that there was a higher coverage of SRH services such as access to condoms and contraceptives among AGYW who dropped out of school compared with those still in school or who graduated. However, those who were still in school or had graduated were more likely to access the combination prevention intervention compared with those who had dropped out. Reaching in school students with the provision of condoms and contraception has been met with resistance, based on the belief that it would encourage sexual activity [54]. The combination prevention intervention reached this population with an intervention that involved assessing their SRH risk and linking them with relevant services [36]. Children should have access to health services in schools. The Global School Health Initiative which aimed to strengthen health promotion in schools was launched in 1995 by WHO [55]. They recognized the potential for schools to play a role in the pairing of children with health services. However, the quality and coverage of health services in schools vary globally.

In relation to the second objective of this study, participants with high absenteeism reported riskier sexual behavior compared with those with low absenteeism. Another study in New York among grade 10 students also found that high absenteeism is associated with risky sexual behavior [56].

Of those with high absenteeism, 10.2% were HIV positive versus only 5.5% of those with low absenteeism (p < 0.05). Bivariate analysis also revealed that AGYW who were still in school and were HIV negative were less likely to be absent from school frequently compared to HIV positive AGYW. A study conducted in South Africa and two studies in Zimbabwe found similar results among young women who had dropped out having had three or more times the odds of HIV infection compared to those who attended school [24, 57, 58]. Although this study was not related to absenteeism, studies have shown that youth who have high or chronic absenteeism are at a high risk for dropping out of school permanently [59]. These findings support the theory mentioned earlier that attending school where there is a safer sexual network and time constraints reduces risky sexual behavior which then reduces HIV infection[60].

Ever having been pregnant was found to be associated with high absenteeism in this study as well as in another study in South Africa [46]. However, during our bivariate analysis, pregnancy did not seem to have as strong of an effect on absenteeism as it did on dropout.

AGYW with high absenteeism rates tended to experience more IPV and rape than those with low absenteeism, with bivariate analysis showing that participants who had been raped were almost two times more likely to have high absenteeism compared to those who had never been raped. A study conducted in the US among adolescents aged 11–15 years also found that reports of previous violence (including IPV) was associated with frequent absenteeism [61]. Another recent study in the US found that adolescents reporting past year dating violence had high odds of absenteeism due to feeling unsafe [62]. This could explain our finding as well especially if the perpetrator was at the same school.

Our study found that those with low school absenteeism had a lower prevalence of having high alcohol use compared to AGYW who were absent frequently. A study conducted in Taiwan found that the risk of truancy was 2.5 times greater after youth had started alcohol or tobacco [27]. Two reviews, one meta-analytic review of 75 studies [14] and another integrative literature review of 37 articles [63] also found that substance abuse was a risk factor for school absenteeism. Some authors found that truancy or being absent from school provided adolescence with free time for delinquent behaviors [64].

We found that there was higher coverage of access to SRH services among AGYW who had high absenteeism compared with those who did not access these services. These results are similar to the findings of those who dropped out of school. It appears that those not in school regularly or at all are more likely to access SRH services. No associations were found between absenteeism and participation in the combination intervention. This means that if the adolescent girls were absent from school, it did not affect their participation in the combination intervention. This could be because of combination intervention implementation arrangements which reached all those registered in school and did not prejudice those who were often absent.

### Strengths and Limitations

A study limitation is that we did not have information about when the dropout occurred, and whether the SRH risks were experienced before or after dropout. The measure for SES has not been validated. Given the cross-sectional study design, we cannot conclude whether the SRH risk and intervention coverage indicators associated with dropout and absenteeism were antecedents to, or consequences of dropout and absenteeism. We do not have data regarding the response rates of those who dropped out versus those who did not and those with high absenteeism versus low absenteeism. A strength of this study is that it is community-based rather than school-based, which enabled us to access and describe the SRH risks and SRH intervention coverage among both those who had and had not dropped out of school. We also were able to access the population of adolescents still registered in school but with high levels of absenteeism, who might not be captured in school-based studies. Despite putting in place procedures for participants for self-completion of questions about sexuality in a private location where the participant felt comfortable, underreporting, and social desirability bias may have affected the findings. It is also possible that the lower rates of SRH service uptake in the low-absenteeism or non-dropout group is due to AGYW not seeing the need for these services because they are not sexually active. We acknowledge that there are likely bidirectional relationships between dropout and absenteeism as the outcomes and the SRH indicators as exposures and the cross-sectional data do not support establishing temporality.

## Conclusion

AGYW who were most at risk of adverse SRH outcomes had higher levels of coverage of most SRH services, which is appropriate. However, compared with AGYW who had dropped out, those in school or who completed school reported lower but relatively high levels of SRH risk and therefore need SRH services. Yet they have less access to the SRH services that are routinely provided except for the services provided through the combination prevention intervention. This demonstrates the value of interventions of this nature which need to be implemented on a wider scale and sustained beyond donor commitments to ensure coverage of SRH interventions across all populations of AGYW, whether or not they are in school. The knowledge generated by our study can inform interventions and services to ensure SRH intervention coverage among adolescents who most need services and interventions.
